# Augmentation of Atrazine biodegradation by two *Bacilli* immobilized on α-Fe_2_O_3_ magnetic nanoparticles

**DOI:** 10.1038/s41598-018-36296-1

**Published:** 2018-12-13

**Authors:** Hina Khatoon, J. P. N. Rai

**Affiliations:** Department of Environmental Science, G.B. Pant University of Agriculture and Technology, Pantnagar (U.S. Nagar), Uttarakhand India

## Abstract

In this study, a novel immobilizing carrier with α-Fe_2_O_3_ magnetic nanoparticles was developed and used for immobilization of atrazine-degrading bacterial isolates of *Bacillus spp*. Since the free cells of microorganisms generally not succeed to degrade pollutants; thus, extra treatments are alluring to make strides biodegradation. Scanning electron microscope (SEM) images appeared that after immobilization the bacterial cells were totally retained and entirely distributed on the surface of α-Fe_2_O_3_ magnetic nanoparticles_._ The performance of α-Fe_2_O_3_ immobilized cells in atrazine (ATZ) degradation was compared with the free cells, which was about 90.56% in 20 days. Experimental results exhibited that ATZ could be degraded at a broad range of physicochemical parameters viz. pH (4.0 to 9.0), temperature (20 to 45 °C), ATZ concentration (50 to 300 mg L^−1^) and agitation speed (50 to 300 rpm), which underlines that α-Fe_2_O_3_ immobilized cells could tolerate a higher range of ATZ concentration as compared to free cells. This research demonstrated that α-Fe_2_O_3_ could be applied as a potential carrier in cell immobilization and biodegradation of ATZ herbicide with greater efficiency.

## Introduction

Atrazine [2-chloro-4-(ethylamino)-6-(isopropylamino)-1,3,5-triazine], a subsidiary of the S-triazines is the most regularly utilized herbicide for controlling broad-leaf and lush weeds in sugarcane, maize, sorghum and other crops across the world. It is very diligent in an impartial environment that makes toxicity to various living organisms like algae, aquatic plants, insects, fishes, and mammals^1,2^. The persistence and mobility of ATZ in the soil are found to be very high, so, it has frequently been identified in surface and groundwater at concentrations well over the reasonable limits and hence considered as a potential environmental contaminant and absolutely considered as most noticeable and awful groundwater contaminant^3^. Amongst the predominant physical, chemical, and biological strategies to expel ATZ from soil and water^4^, the biological processes have been preferred over others^5–7^. Compared with conservative procedures, it is permanent, cost-effective, eco-friendly and nonintrusive to the common biological system^8,9^. Therefore, in both industry and academia, the biodegradation of toxicants at ecologically related concentrations draws utmost interest^10^.

Most recent researches have demonstrated that various microorganisms are able to convert herbicides into nontoxic inorganic compounds^11,12^. The degradation of certain toxic pollutants by various bacterial species, such as *Pseudomonas spp., Burkholderia pickettii, Klebsiella pneumoniae, and Comamonas spp*. have also been reported^13,14^. However, there are some limitations to use free cells in bioremediation, which includes low biodegradation rate, cells separation and substrate inhibition^15,16^. To overcome from these issues, immobilized cell technology can be applied for long-term stabilization of different contaminant remediating microorganisms^17–19^. In general, the implementation of immobilized preparations is done with both properties i.e. the support material and the enzyme, which would largely recover the stability of the immobilized material^20^.

Researchers used alginate and polyvinyl alcohol (PVA) as gel entrapment in the process of cell immobilization. But these methods have some disadvantages which include, the limited transport of substrates and the mechanical fragility of retaining materials in response to environmental changes^21,22^. The Fe_3_O_4_ magnetic nanoparticles could enhance induction of the degrading enzymes, increase enzymes activity and enhance membrane permeability^23^ and Fe_3_O_4_ magnetic nanoparticles also used as immobilizing support as well as for degradation^24^. To the best of our knowledge, the α-Fe_2_O_3_ immobilizing material with the modifications of structural and morphological characteristics of α-Fe_2_O_3_ magnetic nanoparticles after bacterial adhesion has not been reported in the literature. Therefore, the understanding of the morphological and structural characterization of α-Fe_2_O_3_ magnetic nanoparticles and its bacterial cell immobilizing properties is of great significance. Hence, in the present study, the two ATZ degrading bacterial isolates of *Bacillus spp*. i.e. *B. badius* and *B. ensimensis* were immobilized and degradation of ATZ by free and α-Fe_2_O_3_ immobilized cells in varied environmental conditions were examined, which could pave way to improve biodegradation efficiencies.

## Results

### Isolation and Characterization of ATZ Degrading Bacterial Strain

Atrazine degrading bacterial strain ABP6 and ABP8 were isolated from the enrichment culture. Both isolates were gram-positive, aerobic, spore-forming, and rod-shaped. The bacterial isolates ABP6 and ABP8 were tested for 24 different substrates (24 carbohydrates as a carbon source and for two enzymes oxidase and catalase) to study their metabolizing abilities using KB009 part A and B HiCarbohydrateTM Kit. All these biochemical tests were based on the principle of substrate utilization and pH change. ABP6 was positive for oxidase, catalase, and 14 carbohydrates, while ABP8 was positive for oxidase, catalase, and 24 carbohydrates. Table 1 represents the biochemical characteristics of both bacterial isolates.

**Table 1:** Biochemical characteristics of Atrazine degrading both bacterial isolates namely *B. badius* and *B. encimensis* with Biochemical test kits Part (A) and Part (B).

Part (A)	*B. badius* ABP6	*B. encimensis* ABP8	Part (B)	*B. badius* ABP6	*B. encimensis* ABP8
Lactose	+	+	Inulin	+	+
Xylose	−	+	Sodium gluconate	−	+
Maltose	−	+	Glycerol	−	+
Fructose	+	+	Salicin	+	+
Dextrose	+	+	Glucosamine	+	+
Galactose	−	+	Dulcitol	+	+
Raffinose	−	+	Inocitol	+	+
Trehalose	+	+	Sorbitol	+	+
Melibiose	−	+	Mannitol	+	+
Sucrose	+	+	Adonitol	+	+
L-Arabinose	−	+	a-Methyl-D-glucoside	+	+
Mannose	−	+	Ribose	−	+
Catalase	+	+	Oxidase	+	+

(+) sign represents positive reaction, (−) sign represents negative reaction.

### Determination of Atrazine MIC for Bacterial Growth

The minimum inhibitory concentration(MIC) was determined on the basis of bacterial growth on mineral salt agar plates and minimal broth supplemented with atrazine at different concentrations (50, 100, 150, 200, 250, 300 and 350 mg L^−1^) for both bacterial isolates. Atrazine concentration of 300 mg L^−1^ was found to exhibit MIC, after that no growth of bacterial cells was observed. Both bacterial isolates exhibited fast growth up to 200 mg L^−1^ atrazine concentration and also used atrazine as the sole source of carbon and nitrogen. Therefore, this concentration was used in forthcoming experiments on atrazine degradation.

### 16S rRNA Gene Sequencing of bacterial strains

**Figure 1:**
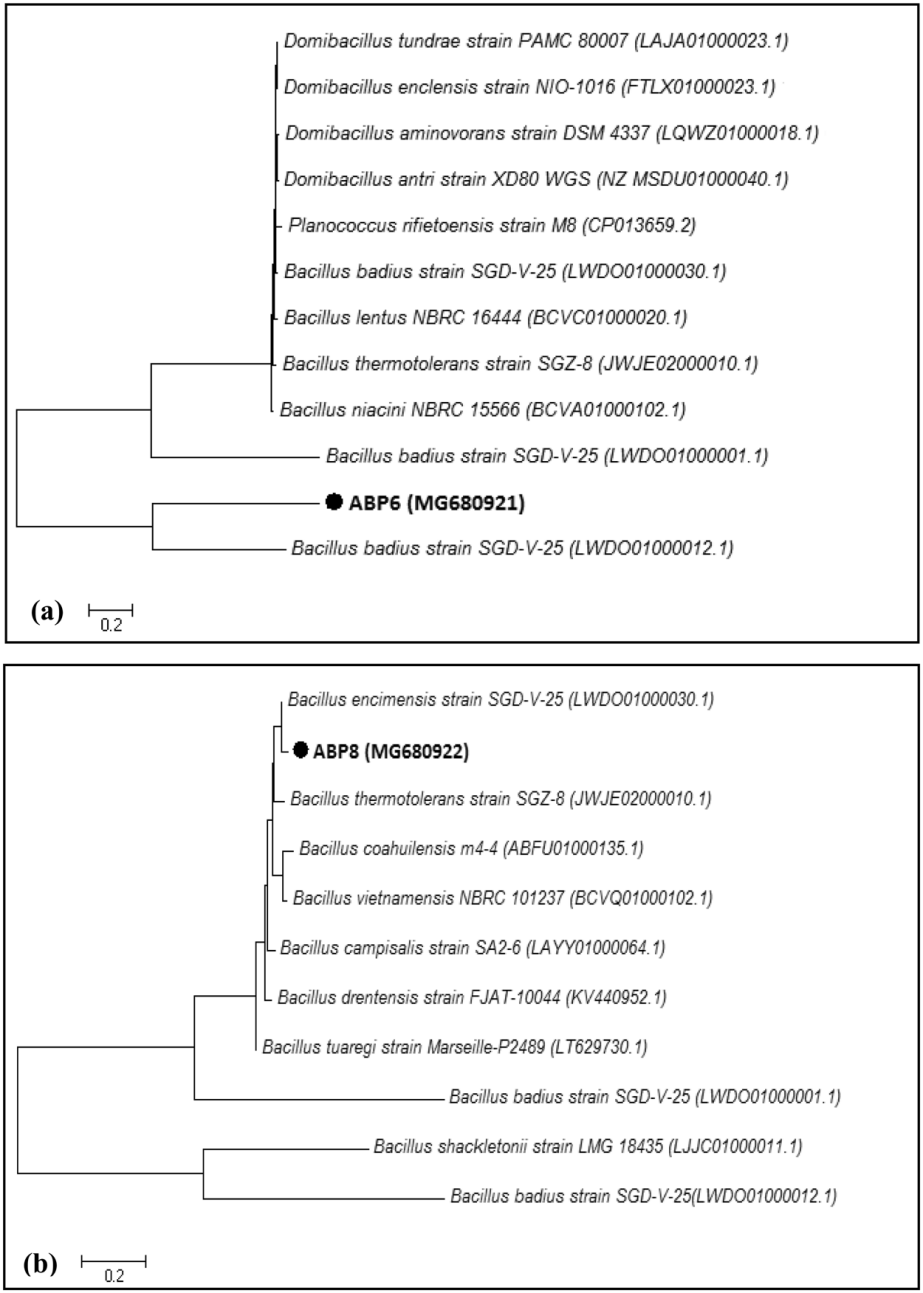
Phylogenetic trees of *B. badius* ABP6 **(a)** and *B. encimensis* ABP8 (**b**) based on the 16S rRNA sequence alignments. Sequences were aligned using Clustal W, distances were calculated using the Kimura 2 parameter method. The trees were built using the neighbour joining method.

16S rRNA partial nucleotide sequence analysis of bacterial isolate was carried out by Chromous Biotech Pvt. Ltd., Bangalore, India. Alignment of the 16S rRNA partial gene sequence of bacterial isolates was performed with sequences present in the GenBank database using BLAST (http://www.ncbi.nlm.nih.gov/blast/). A MEGA version 7.0 software package and neighbor-joining (NJ) method were used for phylogenetic analysis. Submission of the sequence was completed in the GenBank database with accession number MG680921 and MG680922. The bacterial isolates were designated to be *B. badius* ABP6 and *B. encimensis* ABP8. The constructed phylogenetic trees for both bacterial isolates are represented in Fig. 1a,b.

### Analysis of prepared α-Fe_2_O_3_ magnetic nanoparticles

**Figure 2:**
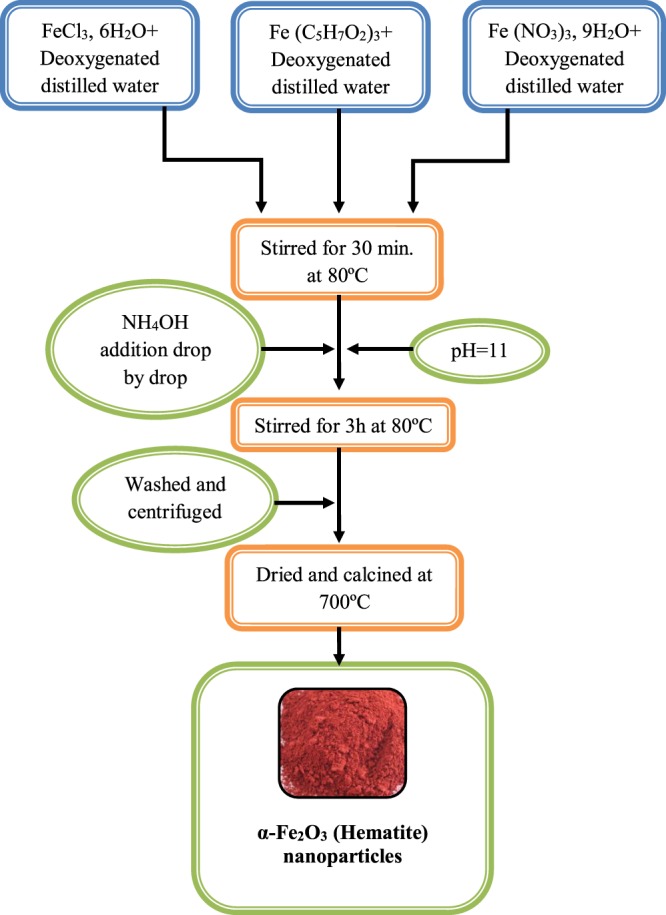
Flowchart for the synthesis of hematite (α-Fe_2_O_3_) nanoparticles by chemical precipitation method.

The α-Fe_2_O_3_ magnetic nanoparticles were synthesized by Chemical precipitation method^25,26^ (Fig. 2) and prepared magnetic (Hematite) nanoparticles were characterized on the basis of XRD, FTIR, UV-Visible Spectra analysis as well as with SEM images.

### XRD Analysis

**Figure 3:**
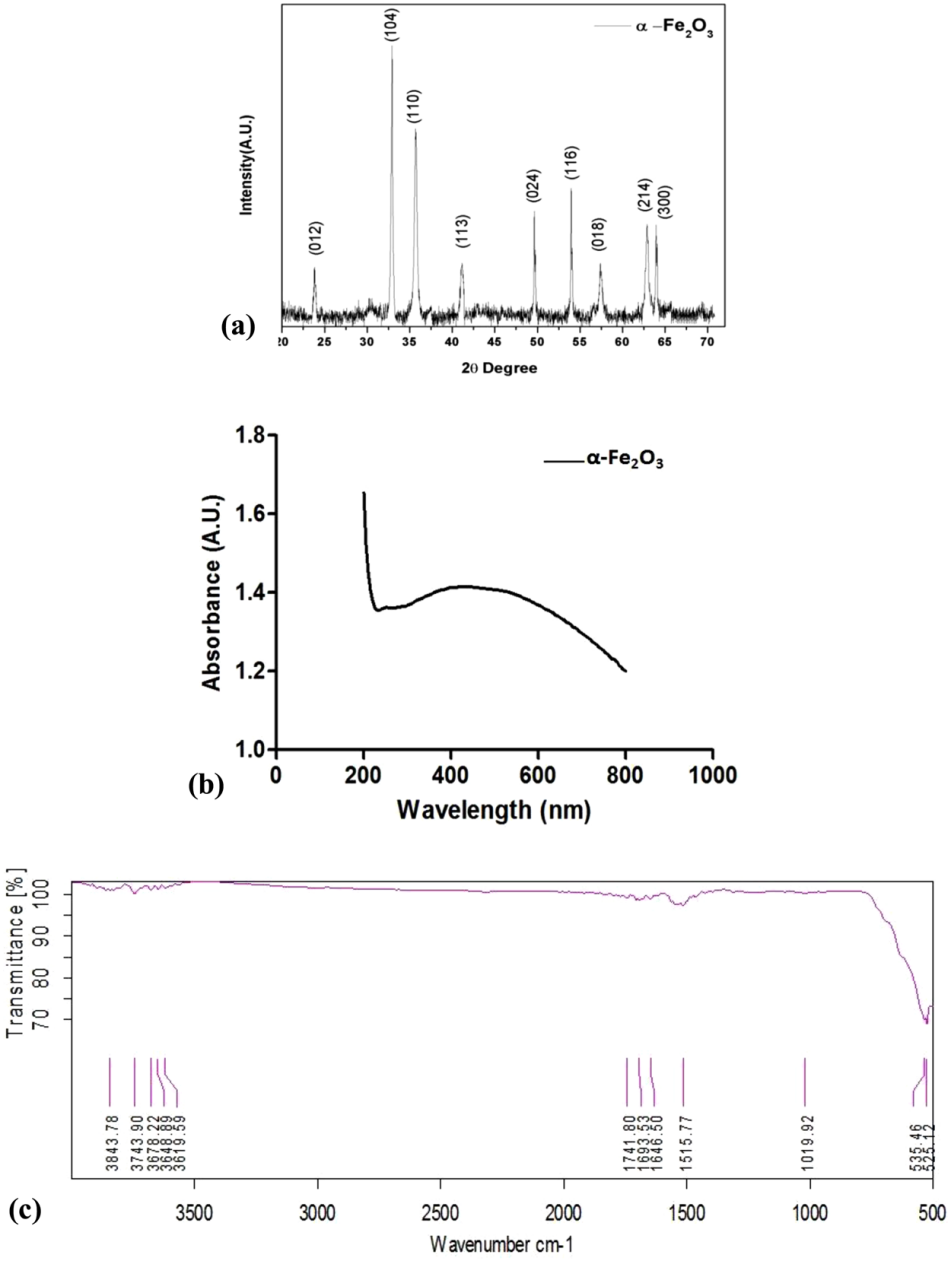
Characterization of chemically synthesized α-Fe_2_O_3_ nanoparticle: (**a**) XRD spectra (**b**) UV–Vis spectrum (**c**) FT-IR Spectrum of synthesized nanoparticle indicating the wave number of the main vibration modes.

The XRD pattern of α-Fe_2_O_3_ magnetic nanoparticles is presented in Fig. 3a. In the present study, the crystalline size of prepared nanoparticles was found to be approximately 7 nm using scherrer’s formula:

$$D=\frac{k\lambda }{\beta \,\cos (\theta )}$$

Here λ is the x-ray wavelength (Cu-Kα, λ = 1.54 Ǻ), k is the machine constant (0.9), β is the full width at half maximum (FWHM in radian) of the peak and θ is the peak angle.

The most intense XRD peak was obtained at 2θ = 32.89°. All the peaks corresponding to the planes (0 1 2), (1 0 4), (1 1 0), (1 1 3) and other planes are found to be matching with the reported information in JCPDS card no.-36-0664 confirming the alpha phase of synthesized iron oxide nanoparticles. Few low intense peaks present in the XRD curve shows the presence of other iron oxide phases in the samples (Fig. 3a). This XRD pattern for α-Fe_2_O_3_ magnetic nanoparticles was also compared with other studies^25,26^ and confirmed as α-Fe_2_O_3_ magnetic nanoparticles peaks in XRD spectrum.

### UV–Visible analysis

The absorption spectra in the UV–Vis range of hematite (α-Fe_2_O_3_) synthesized through chemical precipitation method shows that the absorption curve exhibit an intense absorption in the range of 400–800 nm wavelength. In Fig. 3b, the spectrum of UV-Vis spectroscopy for prepared nanoparticles characterization is shown. This result is consistent with observations of other studies and confirmed as hematite (α-Fe_2_O_3_) nanoparticles absorption spectra in UV-Visible range^25–29^.

### Fourier Transform Infra-Red (FT-IR) spectroscopy

The formation of α-Fe_2_O_3_ magnetic nanoparticles (hematite) was further confirmed by FT-IR spectroscopy. The FT-IR spectrum of hematite is shown in Fig. 3c. The strong absorption peaks at 535 and 525 cm^−1^ can be recognized to the Fe–O band vibrations for α-Fe_2_O_3_ nanoparticles^25,26,30,31^. The very broad absorption band centered at 3743 cm^−1^ and reaching a peak at 1646 cm^−1^ is assigned to the stretching and bending vibrations of the hydroxyl groups and/or water molecules^32^, respectively. In addition, there is a peak at 1515 cm^−1^ that is assigned to the deformation of CH_3_.

### Morphological observation of α-Fe_2_O_3_ nanoparticles, free and immobilized bacterial cells

**Figure 4:**
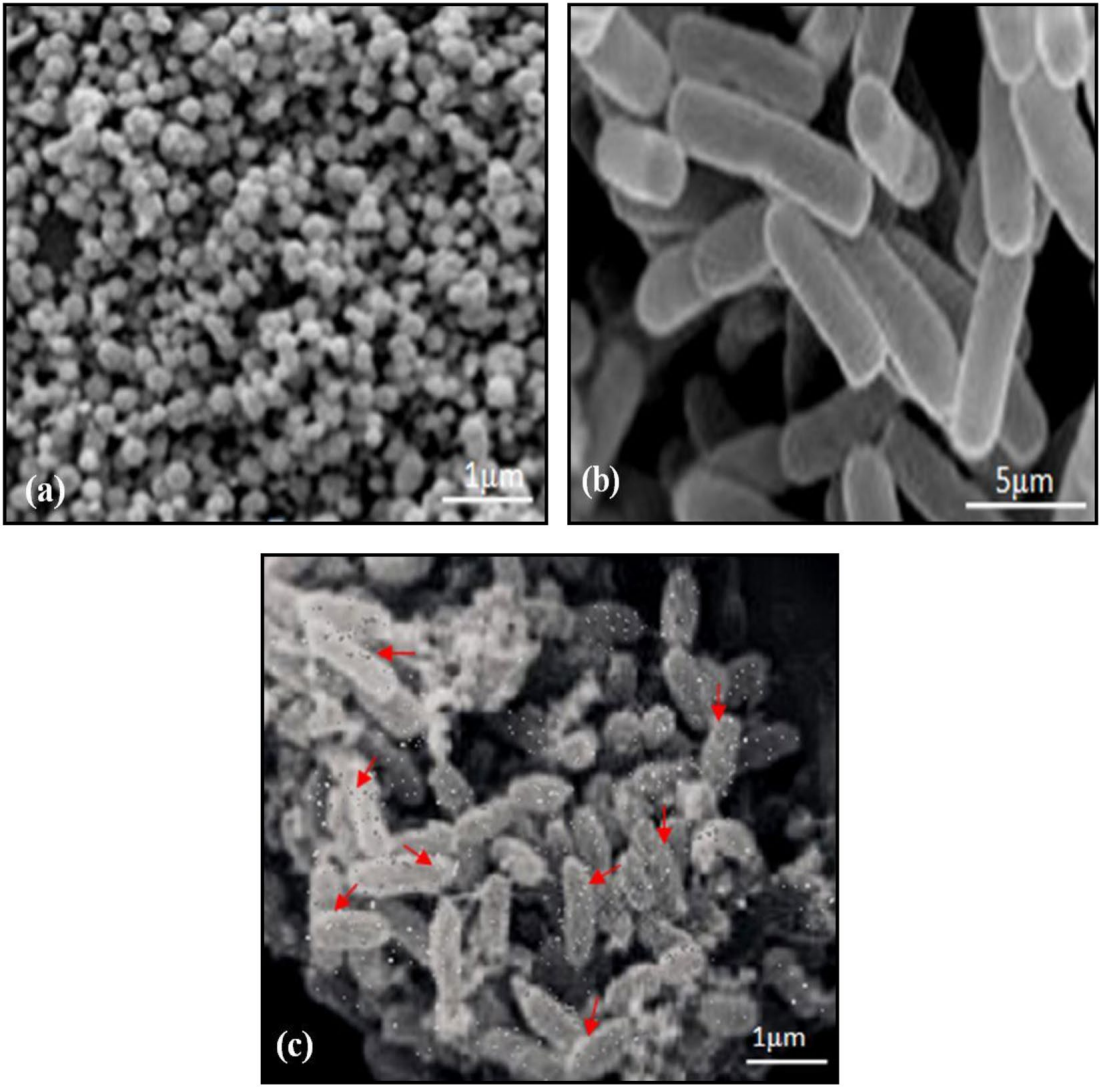
SEM images shows (**a**) The α-Fe_2_O_3_ nanoparticles: before immobilization, (**b**) Free bacterial cells (**c**) α-Fe_2_O_3_ nanoparticle after immobilization with bacterial cells and the red arrows point the locations of α-Fe_2_O_3_ nanoparticles adhesion with bacterial cells.

The surface morphologies of the prepared samples (pure α-Fe_2_O_3_ nanoparticles, free bacterial cells, and immobilized bacterial cells) were studied using a Scanning Electron Microscope. The scanning images of α-Fe_2_O_3_ nanoparticles before immobilization were presented in Fig. 4a, where the shape of initial α-Fe_2_O_3_ magnetic nanoparticles was spherical, irregular columnar, which provides a large area for free bacteria to get in touch with the α-Fe_2_O_3_ surface. Figure 4b represents the scanning image of free bacterial cells (*B. badius* and *B. encimensis* consortium). Figure 4c represents α-Fe_2_O_3_ nanoparticles and bacterial cells after immobilization, where all the bacterial cells were fully immobilized with nanoparticles and formed a bacterial-α-Fe_2_O_3_ immobilized composite. α-Fe_2_O_3_ nanoparticles served as a support material for immobilization of bacteria, because of its size, shape, surface functional groups and natural porous structure, which provides large surface area and high loading volume for bacterial cell adsorption.

### Effects of reaction conditions on ATZ degradation

**Figure 5:**
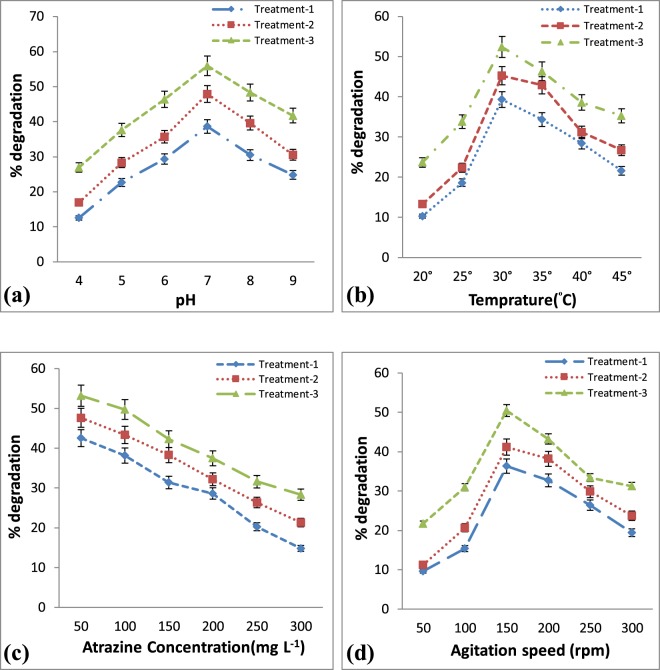
Effects of (**a**) pH, (**b**) Temperature (°C), (**c**) Atrazine concentrations (mg L^−1^) and (**d**) agitation speed (rpm) on biodegradation of atrazine by free and immobilized bacterial cells. (Treatment-1: α-Fe_2_O_3_ without bacterial cells, Treatment-2: free bacterial cells, Treatment-3: α-Fe_2_O_3_ immobilized bacterial cells). Error bars represent standard deviation of triplicate tests and Values are means of three replicates with standard deviation.

The reaction conditions that might influence the ATZ-degrading activity of the microbial composite were explored for four experiments: pH, temperature, atrazine concentration and agitation speed with different treatments (Treatment-1: α-Fe_2_O_3_ without bacterial cells, Treatment-2: free bacterial cells, Treatment-3: α-Fe_2_O_3_ immobilized bacterial cells). Figure 5, shows the optimal pH, temperature, atrazine concentration and agitation speed for degradation in all treatments were 7.0, 30 °C, 200 mg L^−1^ and 150 rpm respectively. Figure 5a revealed that the capability of α-Fe_2_O_3_ without bacterial cells and free bacterial cells on the degradation of atrazine decreased significantly when the pH shifted toward the alkaline or acidic conditions. In contrast, the degradation of atrazine by α-Fe_2_O_3_ immobilized cells changed slowly and maintained at a high level in the whole pH range of 4–9 (>50%) after 10 days of incubation period. Figure 5b depicted the effect of temperature variations on atrazine degradation. At 30 °C temperature, both free and immobilized bacterial cells displayed increasing atrazine degradation. With the further increase in temperature (>30 °C), the atrazine-degradation declined. Nevertheless, Fig. 5b shows that for all temperature ranges the α-Fe_2_O_3_ immobilized bacterial cells represents a better ability to tolerate at different temperature as compared with treatment 1 and 2. When the temperature reached 45 °C the degradation of atrazine using α-Fe_2_O_3_ immobilized cells (treatment 3) decreased slightly to 35.29 ± 1.9%, but it dropped significantly to 21.62 ± 0.98% and 26.73 ± 1.08% with treatment 1 and 2. The change of percent degradation rate with the increment of ATZ initial concentrations is presented in Fig. 5c, which clearly depicted that all treatments possessed greater degradation ability at low concentration of ATZ i.e. 50 mg L^−1^ and after that decreased as the concentration increased up to 300 mg L^−1^. The effect of different agitation speeds (50 to 300 rpm) on atrazine degradation is represented in Fig. 5d. At 150 rpm, α-Fe_2_O_3_ immobilized bacterial cells (treatment 3) displayed increasing atrazine degradation. The degradation rate decreased significantly as the agitation speed shifted towards higher and lower range after 10 days of incubation period by treatment 1 and 2. These results revealed the superiority of the α-Fe_2_O_3_ immobilized bacterial cells (treatment 3) in pH and thermal stability as compared to the α-Fe_2_O_3_ without bacterial cells and free bacterial cells (treatment 1 and 2).

### The viability of bacterial cells before and after immobilization

The stability of immobilized as well as free bacterial cells was checked with viability count (OD600 and CFU mL^−1^), and the required data is represented in Table 2, which showed growth and viability of bacterial cells at different incubation period before and after immobilization, to quantify the immobilized cells on magnetic nanoparticles, as well as free bacterial cells. Results exhibited that immobilized bacterial cells have the better capability as compare to free cells and their growth has been increased as the incubation period increased. At earlier incubation period (0 days) the growth and viability for both the bacterial cells were found to be same, but as the incubation period increased from 0 to 15 days, the growth and viability were also increased and after 15 days of incubation, the growth of both free and immobilized bacterial cells declined gradually.

**Table 2:** The growth of Free bacterial cells and immobilized bacterial cells in Mineral salt broth medium at different optimized conditions (pH: 7, Temprature: 30 °C, Atrazine Concentration: 100 mg L^−1^) up to 25 days of incubation period.

Time (days)	Free bacterial cells		Immobilized bacterial cells	
	Value of OD600	Bacteria counts (CFU mL^−1^)	Value of OD600	Bacteria counts (CFU mL^−1^)
0	0.53 ± 1.6b	2.7 × 10^6^	0.53 ± 2.2b	2.7 × 10^6^
5	0.57 ± 2.5a	7.8 × 10^6^	0.62 ± 1.7a	3.4 × 10^7^
10	0.64 ± 1.3b	4.1 × 10^7^	0.70 ± 1.5c	2.7 × 10^8^
15	0.67 ± 1.8b	5.6 × 10^7^	0.78 ± 2.4a	3.5 × 10^8^
20	0.64 ± 2.1a	3.9 × 10^6^	0.76 ± 1.2c	4.8 × 10^7^
25	0.56 ± 1.4c	6.5 × 10^5^	0.67 ± 0.9b	7.6 × 10^6^

The data presented are means of three replicates with standard deviation. Different letters indicate significant differences (p < 0.05, LSD test).

### Atrazine biodegradation by both free and immobilized cells

**Figure 6:**
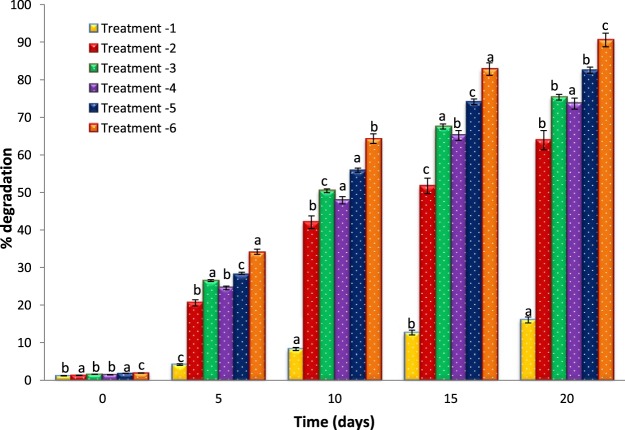
Percent degradation of atrazine by free and immobilized bacterial cells with different treatments. (Treatment 1: Control-MSM + Atrazine, Treatment 2: MSM + Atrazine + α-Fe_2_O_3_, Treatment 3: MSM + Atrazine + *B. badius*, Treatment 4: MSM + Atrazine + *B. encimensis*, Treatment 5: MSM + Atrazine + free bacterial consortium, Treatment 6: MSM + Atrazine + α-Fe_2_O_3_ immobilized bacterial consortium). Error bars represent standard deviation of triplicate tests and Values are means of three replicates with standard deviation.

As shown in Fig. 6 and Table 3, the fastest biodegradation of ATZ occurred with the α-Fe_2_O_3_ immobilized bacterial cells (Treatment 6). The percent of ATZ degradation reached 34.21 ± 1.03% after 5 days, and 64.32 ± 1.33% after 10 days. Whereas, 90.56 ± 1.69% ATZ degradation was achieved after 20 days of incubation period, using α-Fe_2_O_3_ immobilized cells. The rate of ATZ degradation by immobilized bacterial isolate enhanced with the increase of incubation time i.e. from 0 to 20 days. As a result ATZ residues for immobilized cells were found almost negligible after 20 days.

**Table 3:** Recovered atrazine concentration after degradation by free and immobilized bacterial cells with different treatments (Remaining ATZ concentration after degradation).

	Incubtion period (days)				
	0	5	10	15	20
Treatment -1 (Control:MSM + Atrazine)	198.77 ± 0.99b	195.83 ± 1.05c	191.69 ± 1.25a	187.31 ± 1.34b	183.96 ± 1.12a
Treatment -2 (MSM + Atrazine + α-Fe_2_O_3_)	198.68 ± 0.87a	179.41 ± 1.13b	157.93 ± 1.09b	148.27 ± 1.61c	136.09 ± 1.38b
Treatment -3 (MSM + Atrazine + *B.badius)*	198.39 ± 1.06b	173.48 ± 1.48a	149.56 ± 0.93c	132.44 ± 1.21a	124.67 ± 1.07b
Treatment -4 (MSM + Atrazine + *B.encimensis)*	198.52 ± 0.84b	175.42 ± 1.07b	152.09 ± 0.99a	134.83 ± 1.53b	126.38 ± 0.97a
Treatment -5 (MSM + Atrazine + *B.badius* + *B.encimensis)*	198.13 ± 0.79a	171.52 ± 0.95c	144.07 ± 1.02a	125.88 ± 1.18c	117.48 ± 1.05b
Treatment -6 (MSM + Atrazine + α-Fe_2_O_3_ immobilized bacterial cells)	198.04 ± 0.96c	165.79 ± 1.03a	135.68 ± 1.33b	115.18 ± 1.86a	109.44 ± 1.69c

(Values are written in mg L^−1^, Initial ATZ concentration = 200 mg L^−1^).

The data presented are means of three replicates with standard deviation. Different letters indicate significant differences (p < 0.05, LSD test).

Whilst, with other treatments (1–5) the ATZ degradation rate was lowest as compared to immobilized cells in different incubation periods.

## Discussion

Generally, herbicides applied in culturable lands are included in sorption, desorption, transport, volatilization, and alteration processes^33,34^. Based on the understanding of these physicochemical or biochemical behaviors, superior procedures could be got for compelling utilization of dynamic compounds as well as remediation of herbicide-contaminated locales. Biodegradation includes the utilization of living microorganisms or their enzymes to detoxify pollutants, which has been by and large considered as a successful and cost-effective method for the removal of contaminants^35^. It was emphasized that different microbes had the capacity of efficiently degrading ATZ including *Chelatobacter heintzii*^36^, *Enterobacter (E. cloacae), Bacillus (B. cereus and B. anthracis), Pseudomonas (P. aeruginosa, P. balearica, P. indica and P. otitidis), Ochrobactrum (O. intermedium) and Providencia (P. vermicola)*^37^ and *P. fluorescence* + *P. putida*^38^. In Present study, two exceedingly successful strain of *Bacillus spp*. (*Bacillus badius* ABP6 *and Bacillus encimensis* ABP8) used for ATZ degradation were able to utilized ATZ as carbon and nitrogen source. While comparing to the treatments with higher introductory concentrations, the degradation at lower concentrations was more noteworthy and proficient; this empowered the strain to operate in circumstances with low residues. Prior studies have demonstrated that generally low levels of herbicide residues would influence and cause a chain of impacts on the biological system^39,40^.

The obtained results indicated that the bacterial cells immobilized on α-Fe_2_O_3_ magnetic nanoparticles have greater ability to tolerate under high acidic and alkaline conditions as compared to free bacterial cells. The bacterial cell diversity, their enzyme activity as well as nutrient solubility get affected by the pH of culture medium^41,42^. The immobilization of cells has a better capacity to stabilize inner pH value of microbes and which might have led to best external conditions and favorable environment for microorganisms as compared to the free bacterial cells with better tolerance under fluctuating conditions of pH^24,41–46^. In the optimization of temperature, results showed that both free and immobilized bacterial cells have reduced capability to degrade atrazine at a higher temperature. This is due to the fact that higher temperature has a negative effect on the activity of the bacteria and would cause a decline in oxygen solubility, hence, hindered its biodegradation capabilities^16^. After checking the stability of free and immobilized bacterial cells, it was clear that immobilized cells have better growth, viability and higher activity as compared to free cells, which ultimately leads to increase the biodegradability of contaminants by immobilized cells. The surface charge, species/strain, growth phase, growth rate, surface structure and extracellular polymeric substances of bacterial cells could also affect the immobilization process for physicochemical interactions with nanoparticles. In the present experiment, the magnetic nanoparticles were used only for the adsorption of bacterial cells, they do not affect the growth and viability of bacterial cells during immobilization; but they can reduce the bacterial growth when the concentration of nanoparticles reached above the permissible limit^24,42–47^. In feasible application, the degradation implementation was not stable and compelling as tried in research facility since the coordinate utilizes of free cells was affected by numerous variables. Utilizing diverse immobilized microbes may give a conceivable arrangement, which has been demonstrated tediously as a compelling approach for contaminants remediation^13,14^. It was reported that the movement of the immobilized composite is immensely influenced by the properties of support material^16^. Iron oxide nanoparticles (IONs), with their unique magnetic properties, high surface/volume ratio, surface functional groups, size, shape and superior biocompatibility, showed great promise for applications in bioprocesses^47–54^. Therefore, choosing a steady and economical support with great biocompatibility is the key to the immobilized composite and overall results revealed an advantage of α-Fe_2_O_3_ immobilized cells on ATZ degradation over free cells. This attribute further signifies the efficient performance of bacterial isolates and α-Fe_2_O_3_ magnetic nanoparticles immobilizing support. SEM images represented that bacterial cells completely adhered on the surface of α-Fe_2_O_3_ magnetic nanoparticles, and their degrading movement held after immobilization. This statement has been explained in light of the study made by others^42,47^. Moreover, it is accepted that the immobilization technique should be a generally basic process that does not require a profoundly unadulterated enzyme preparation or a costly support that may not be commercially accessible^15^.

The present study demonstrated that the use of combined α-Fe_2_O_3_ magnetic nanoparticles in conjunction with indigenous soil microbes could substantially enhance the degradation efficiency of ATZ compared with the individual treatments. In addition, the experimental data suggested that the immobilized α-Fe_2_O_3_ magnetic nanoparticles could significantly improve soil microbial populations and enzyme activities, which in turn resulted in improved degradation efficiency of *Bacillus spp*.

## Conclusion

Utilization of dynamic microorganisms is an efficient and economical strategy, while microbial immobilization gives a breakthrough in the restricted application of microbes in *in-situ*. In the present study, α-Fe_2_O_3_ was utilized in herbicide biodegradation as fabulous stacking supports for bacterial immobilization. Compared with freely suspended cells and immobilized cells, the efficiency of ATZ degradation by α-Fe_2_O_3_ immobilized cells was improved significantly. On the basis of results obtained, it can be concluded that α-Fe_2_O_3_ has much potential application as a carrier in cell immobilization. However, it also requires fine tuning at pilot scale experiments.

## Methods

### Chemicals

Analytical grade Atrazine (>98% purity) and methanol were obtained from Sigma-Aldrich, USA. ATZ was dissolved in methanol at a stock concentration of 100 mg L^−1^ and stored at 4 °C prior to use. All reagents used in the synthesis procedure were an analytical grade of Himedia Laboratories, India. Iron (III) chloride hexahydrate (FeCl_3_.6H_2_O) was procured from Sigma-Aldrich, USA, while ammonium hydroxide (NH_4_OH) and ethanol (C_2_H_6_O) from Merck, India.

### Soil sample collection and Isolation of ATZ -degrading bacteria

Soil samples at depth of 15 cm were collected from Norman E. Borlaug, Crop Research Centre, Pantnagar, India, situated at an altitude of 243.84 above mean sea level, 29°N latitude and 79.3°E longitude, after 10 days of herbicide spray in the Maize fields. For the isolation of ATZ-degrading bacteria from contaminated soil, 5 g of Soil samples in Erlenmeyer flasks (250 ml) was added with the minimal broth (50 mL) having 50 mg L^−1^ ATZ concentration. The enrichment was done by incubating at 30 °C on a rotary incubator (150 rpm). After 7 days, the broth culture (5 mL) from flask was reinoculated into fresh minimal media (50 mL) concentrated with 100 mg L^−1^ ATZ under the above-mentioned situations. The same procedure was repeated twice up to 200 mg L^−1^ of ATZ concentration. After that, 0.2 mL of final culture broth was pour plated on agar plates for isolation of a single colony. Each colony, considered as a diverse species, was more than once streaked on agar plates. To obtain a pure culture of best ATZ degradation, 10 times streaking followed screening was done. After the consecutive enrichment culture, bacterial isolates showing prolific growth in ATZ supplemented mineral salt agar medium and designated as ABP6 and ABP8.

### Determination of ATZ Minimum Inhibitory Concentration (MIC)

Minimum inhibitory concentration for bacterial isolates ABP6 and ABP8 was determined to check the toxicity and tolerable limit of atrazine on the basis of growth. Bacterial isolates were streaked on mineral salt agar plates supplemented with atrazine as the sole source of carbon and energy at the concentrations of 50, 100, 150, 200, 300, 350 and 400 mg L^−1^. After that these agar plates were incubated at 30 °C for 72 h, for the appearance of bacterial colonies.

### Identification of ATZ Degrading Bacterial Isolates ABP6 and ABP8

Morphological and Biochemical tests were done for the identification of most active atrazine degrading bacterial isolates ABP6 and ABP8. These strains were tested for 24 different substrates (24 carbon sources and 2 enzyme activities viz. Catalase and Oxidase) and KB009 part A and B HiCarbohydrateTM Kit were used to check their metabolizing abilities (Table 1). These tests were based on the principle of pH change and substrate utilization.

### Synthesis of α-Fe_2_O_3_ Nanoparticles

Hematite nanoparticles (α-Fe_2_O_3_) were synthesized by chemical precipitation method^25,26^. In a typical synthesis procedure, 0.1 M solution was prepared by dissolving iron (III) chloride hexahydrate (FeCl_3_.6H_2_O) in 100 mL of distilled water under vigorous stirring for 45 min at 80 °C. 50 mL aqueous solution of NH_4_OH (2M) was prepared in distilled water. NH_4_OH solution was added dropwise to the solution until the pH reached a value of 11. The solution was stirred at 80 °C for 4 hours to complete the reaction and then centrifuged, washed with distilled water and ethanol. The precipitate was dried in a hot air oven at 80 °C for 6 hours and sintered at 700 °C for 2 hours to get α-Fe_2_O_3_ magnetic nanoparticles (Fig. 2).

### Characterization of chemically synthesized α-Fe_2_O_3_ nanoparticles

Prepared α-Fe_2_O_3_ magnetic nanoparticle’s characterization was done by utilizing various techniques. Purity and Crystalline nature of synthesized nanoparticles were assessed by XRD using an X-ray Diffractometer. The Ultraviolet-Visible (UV–Vis) absorption of the samples was recorded utilizing UV–Vis Spectrophotometer (GENESYS 10S). Fourier Transform Infra-Red (FT-IR) spectra of samples were recorded at transmission range from 4000 to 500 cm^−1^ by FT-IR spectrometer (Alpha 200855). Scanning electron microscope (SEM) was used for examining the morphology of synthesized nanoparticles, free bacterial cells and immobilized bacterial cells with nanoparticles.

### Immobilization of bacterial isolates

Bacterial isolates were immobilized on α-Fe_2_O_3_ magnetic nanoparticles. For the immobilization process, the particles of α-Fe_2_O_3_ were washed with distilled water thrice and dried in an oven at 105 °C for 6 h. The Minimal salt medium (300 mL) was autoclaved at 121 °C for 20 minutes, then after α-Fe_2_O_3_ magnetic nanoparticles (5 g) were added in the sterilized medium. Cell suspension (10.0%, v/v) was inoculated into 500-mL Erlenmeyer flasks containing the autoclaved minimal medium (300 mL) supplemented with 200 mg L^−1^ atrazine. The mixture of bacterial cells and nanoparticles of α-Fe_2_O_3_ was incubated at 30 °C and 150 rpm for 48 h until cells adsorbed on the surface of α-Fe_2_O_3_ to form immobilize composite. The absorbance of the supernatants was determined at 600 nm in order to determine the amount of adsorption. The immobilized cells were washed tenderly twice with distilled water and preserved for prior to use. The morphological characters observation of the immobilized bacterial samples was done with the help of a scanning electron microscope (SEM).

### Optimization of reaction conditions for atrazine degradation

The optimized conditions such as pH (4.0 to 9.0), temperature (20 to 45 °C), ATZ concentration (50 to 300 mg L^−1^) and agitation speed (50 to 300 rpm) for ATZ degradation with different treatments (Treatment-1: α-Fe_2_O_3_ without bacterial cells, Treatment-2: free bacterial cells and Treatment-3: α-Fe_2_O_3_ immobilized bacterial cells) were observed with minimal salt medium during experiment. The single-factor experiment was used for examining the effect of each factor and observed that only the tested factor was changed accordingly. The ATZ residue was measured with HPLC after 10 days of incubation period.

### Growth and Viability of free and immobilized bacterial cells

The viability of bacterial cells with optimized conditions, before and after immobilization process was checked at different incubation periods (0 to 25 days) by optical density at 600 nm (OD600) using UV–Vis Spectrophotometer (Virion Bio 50) and live cell assessment through colony forming unit (CFU) counting on plates^55^ by following formula:

$${\rm{CFU}}=(\frac{{\rm{No}}{\rm{.}}\,{\rm{of}}\,{\rm{colonies}}\times {\rm{dilution}}\,\mathrm{factor}\,}{{\rm{Volumes}}\,{\rm{of}}\,{\rm{the}}\,{\rm{sample}}\,{\rm{taken}}\,({\rm{mL}})})$$

### Biodegradation of ATZ by free and immobilized bacterial cells

A batch experiment was set up for ATZ biodegradation in a 100 mL Erlenmeyer flask containing 50 mL Minimal salt medium (MSM) supplemented with ATZ (200 mg L^−1^) and incubated at 30 °C with a constant agitating speed of 150 rpm on shaking incubator followed by different treatments. Treatment-1 contained MSM (50 mL) and Atrazine (200 mg L^−1^) as control, Treatment-2 contained MSM (50 mL), Atrazine (200 mg L^−1^) and α-Fe_2_O_3_ nanoparticles (0.8 g), Treatment-3 contained MSM (50 mL), Atrazine (200 mg L^−1^) and *B. badius* (10.0%, v/v), Treatment-4 contained MSM (50 mL), Atrazine (200 mg L^−1^) and *B. encimensis* (10.0%, v/v), Treatment-5: MSM (50 mL), Atrazine (200 mg L^−1^), *B. badius* and *B. encimensis* consortium (10.0%, v/v), Treatment-6 contained MSM (50 mL), Atrazine (200 mg L^−1^) and α-Fe_2_O_3_ immobilized bacterial cells. Samples from each treatment were withdrawn at intervals of 0, 5, 10, 15 and 20 days and used for the analysis of residual ATZ concentration. Extraction was done with dichloromethane, and the organic layer was dehydrated, dried, and redissolved in methanol. After filtration, the samples were subjected to high-performance liquid chromatography (HPLC) to determine the degradation of ATZ.

### HPLC analysis of atrazine

The residual ATZ was analyzed on a Dionex ultimate 3000, HPLC equipped with a C18 reversed phase column (250 × 4.6 mm id, 5 μm), and the analytical method is listed in Table 4. Residual ATZ concentration was quantified using a standard curve plotted between detector response (absorbance) and known concentration.

**Table 4:** Reaction conditions for HPLC analysis of remaining atrazine residue after biodegradation by bacterial isolates.

Model:	Dionex ultimate 3000
Column:	C-18 Reverse Phase (250 × 4.6 mm id, 5 μm)
Detector:	UV- detector
Solvent:	Methanol:Water (80:20)
Flow rate:	1 ml/min.
Retention time:	7 min

### Data analysis

All of the experiments were carried out with three sovereign experiments, and the results were the means of three replicates. The percent degradation of ATZ was analyzed according to the following formula:

$${\rm{Percent}}\,( \% )\,{\rm{ATZ}}\,{\rm{degradation}}=\frac{{\rm{Ac}}-{\rm{As}}}{{\rm{Ac}}}\times 100$$

where, As (mg/L) is the residual concentration of sample, and Ac (mg/L) represents the concentration of control sample.

### Statistical analysis

The experimental data were processed for calculating the standard error of the means and multi-factorial analysis of variance as available in the SPSS statistical package (Stat Graphics Plus V. 11) and expressed at 0.05 probability level. The significance (p < 0.05) of differences was treated statistically by one and two-way analysis of variance (ANOVA) and evaluated by post hoc comparison of means using the lowest significant differences (LSD).
